# Very Early Recurrence Following Liver Resection for Intrahepatic Cholangiocarcinoma: Is It Predictable by Clinical Preoperative Factors?

**DOI:** 10.1111/ans.70311

**Published:** 2025-08-28

**Authors:** Francesco Ardito, Francesco Razionale, Andrea Campisi, Çınar Turgay, Alessandro Coppola, Simone Vani, Maria Vellone, Felice Giuliante

**Affiliations:** ^1^ Hepatobiliary Surgery Unit Foundation Policlinico Universitario A. Gemelli, IRCCS Rome Italy; ^2^ Department of Translational Medicine and Surgery Università Cattolica del Sacro Cuore Rome Italy; ^3^ Department of Surgery Sapienza University of Rome Rome Italy

**Keywords:** intrahepatic cholangiocarcinoma, liver resection, overall survival, personalized medicine, prognostic factors, very early recurrence

## Abstract

**Background:**

Approximately one‐quarter of patients undergoing resection for intrahepatic cholangiocarcinoma (ICC) experience very early recurrence (within 6 months after liver resection), which is associated with a poor prognosis. Identifying factors associated with very early recurrence may help optimize patient selection for surgery and avoid futile, high‐risk hepatectomies. The aim of this study was to assess whether preoperative clinical factors alone can reliably predict very early recurrence following curative liver resection for ICC.

**Methods:**

A retrospective analysis was conducted on 83 patients who underwent liver resection between 2010 and 2020.

**Results:**

The 5‐year overall survival (OS) rate for the entire cohort was 51.4%. Recurrence occurred in 54 patients (65.1%), with 17 (20.5%) experiencing very early recurrence. The 5‐year OS for patients with very early recurrence was significantly lower than for those without it (0% vs. 48.7%, respectively; *p* = 0.013). Preoperative clinical prognostic factors failed to identify patients at high risk of very early recurrence, which occurred in 21% of patients classified as low risk.

**Conclusions:**

Preoperative clinical factors alone are insufficient for accurate risk stratification. Integrating clinicopathological data with molecular classifications of ICC is urgently needed to enable a more personalized oncological approach for these patients.

## Graphical abstract

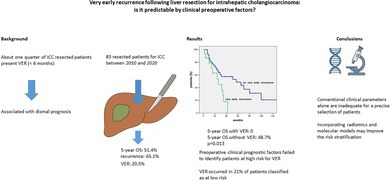

## 1 Introduction

Intrahepatic cholangiocarcinoma (ICC) is the second most common primary liver tumor, accounting for approximately 10%–15% of all primary liver cancers [1]. Its incidence has been steadily increasing worldwide on an annual basis [2, 3].

Currently, complete surgical resection is the only potentially curative treatment for ICC, although it is feasible in only 30%–40% of patients [4]. However, long‐term outcomes after curative liver resection remain poor, with reported 5‐year overall survival rates ranging from 20% to 40% [5, 6, 7]. These disappointing results are largely attributable to the high rate of tumor recurrence following surgery, which occurs in 50%–70% of cases [8, 9, 10].

Several studies have analyzed overall survival based on the timing of recurrence after liver resection [7, 11]. These studies demonstrated that patients experiencing early recurrence (within 24 months) have a worse prognosis and a distinct recurrence pattern compared to those with late recurrence (> 24 months) [7, 11]. However, the commonly used 2‐year cutoff to define early recurrence may not be appropriate for patients undergoing resection for ICC. In fact, the majority of these patients tend to experience recurrence within the first 12 months after surgery [10], and approximately one‐quarter present with very early recurrence (within 6 months) [11]. Very early recurrence is associated with a significantly worse prognosis and a lower likelihood of undergoing repeat resection, raising concerns about the potential futility of liver resection in this subset of patients [12, 13].

For these reasons, recent studies have focused on identifying factors associated with early recurrence (within 1 year) and very early recurrence (within 6 months) following liver resection for ICC [12, 13, 14]. Identifying patients at high risk of very early recurrence may help guide selection for liver resection versus alternative treatment strategies, may help to plan neoadjuvant chemotherapy, and enable the personalization of postoperative surveillance protocols.

The aim of this study was to evaluate whether preoperative clinical factors alone are sufficient to predict recurrence, particularly very early recurrence, after curative liver resection for ICC, and to assess how effectively these factors can be applied in real‐world clinical practice at both individual and institutional levels.

## 2 Materials and Methods

This study is a retrospective observational analysis conducted at a single center. It included patients who underwent liver resection for ICC between January 2010 and December 2020 at our institution. Only patients who underwent curative‐intent resection were included in the analysis. Patients with a final pathological diagnosis of combined primary liver tumors (e.g., hepatocholangiocarcinoma) were excluded. All included patients were evaluated by thoracoabdominal computed tomography (CT) and abdominal magnetic resonance imaging (MRI) [15]. In cases of preoperative suspicion of locoregional lymph node involvement, patients were considered for neoadjuvant chemotherapy with gemcitabine plus cisplatin. Radiological re‐evaluation was performed at 3 and 6 months. In cases of favorable response or stable disease, liver resection was subsequently planned. Criteria for radiologically suspicious regional lymph nodes included a short‐axis diameter greater than 1 cm and the presence of contrast enhancement on preoperative imaging. In cases with radiological suspicion of peritoneal carcinomatosis or distant metastatic lymph node involvement, a PET scan was performed. Liver resections were classified according to the terminology of the International Hepato‐Pancreato‐Biliary Association (IHPBA) [16]. Resections involving three or more liver segments were defined as major hepatectomies. The surgical technique for liver resection adopted in our unit has been previously described [17, 18]. All patients underwent regional lymphadenectomy, which included dissection of the hilar, pericholedochal, hepatic artery, periportal, and superior retropancreatic lymph nodes [19]. Regional lymphadenectomy was routinely performed in all cases, regardless of whether the liver resection was classified as major or minor. The same lymphadenectomy protocol was applied consistently in both open and laparoscopic approaches.

The following data were collected for each patient: demographic information, presence of underlying chronic liver disease, and use of neoadjuvant chemotherapy. The cutoff value for preoperative CA 19‐9 was set at 54 U/mL, based on a recent multicenter study by Wei T. et al. [20], which analyzed 1095 resected patients and identified CA 19‐9 > 54 U/mL as one of the strongest predictors of both overall survival (OS) and disease‐free survival (DFS). Operative details collected included the type of resection, use of pedicle clamping, and intraoperative blood transfusions. Early outcomes encompassed postoperative complications and 90‐day mortality. Postoperative complications were graded according to the Clavien‐Dindo classification system [21], with severe complications defined as Clavien‐Dindo grade ≥ 3. Late outcomes included 5‐year OS and DFS rates.

Tumor staging was performed according to the TNM classification of the UICC staging system, 8th Edition [22]. Pathological data included the presence of perineural invasion, resection margin status, and lymph node involvement. An R0 resection was defined as the absence of microscopic tumor invasion at the resection margin (tumor‐free margin ≥ 1 mm), while R1 resection indicated complete macroscopic removal with microscopic tumor invasion at the margin (tumor‐free margin = 0 mm). Following the classification proposed by Conci et al. [23], tumor nodules were categorized as: single tumor (type I); single tumor with satellite nodules within the same Couinaud liver segment (type II); and multifocal tumors involving different Couinaud liver segments (type III).

Adjuvant chemotherapy with gemcitabine was administered to patients with T3 to T4 tumors or those with lymph node metastases. Follow‐up consisted of serum CA 19‐9 measurement and thoracoabdominal CT scans every 3 months for the first 2 years, then every 6 months for the subsequent 3 years. Time to recurrence was defined as the interval between liver resection and the detection of any recurrence on radiological imaging. Very early recurrence was defined as recurrence occurring within 6 months after liver resection [13, 14]. Early recurrence was defined as recurrence occurring between 6 and 12 months post‐resection, while late recurrence referred to recurrence occurring more than 12 months after surgery.

Based on the paper by Tsilimigras et al. [14], we applied their very early recurrence calculator and scoring system to stratify the prognosis of resected patients in our analysis. Preoperative and postoperative risk scores for the likelihood of very early recurrence were calculated for each patient. The variables included in the preoperative risk score were age, race, presence of cirrhosis, tumor size, number of lesions, and suspicion of positive regional lymph nodes. The postoperative risk score incorporated age, race, tumor size, number of lesions, microvascular invasion, positive regional lymph nodes, and margin status. Patients were classified into three distinct risk groups for very early recurrence (low, intermediate, and high) based on either the preoperative or postoperative scores. According to the Tsilimigras et al. scoring system, patients were stratified into risk groups as follows: for the preoperative score, 0–3 (low risk), 4–5 (intermediate risk), and 6–9 (high risk); for the postoperative score, 0–4 (low risk), 5–6 (intermediate risk), and 7–10 (high risk).

The primary objective of this study was to evaluate whether clinical preoperative factors alone are sufficient to predict recurrence, particularly very early recurrence, following curative liver resection for ICC. The study was conducted in accordance with the Declaration of Helsinki and received approval from the Institutional Review Board of the Catholic University of the Sacred Heart on 17 March 2022 (Prot. n. 0010136/22; ID: 4832). Continuous variables are presented as medians and interquartile ranges (IQR), while categorical variables are expressed as counts and percentages. OS was calculated from the date of liver resection to the date of death or censored at the last follow‐up. Survival curves were generated using the Kaplan–Meier method and compared using the log‐rank test. Multivariable regression analyses were performed to identify independent prognostic factors for OS, recurrence, and very early recurrence. Variables with *p* < 0.2 in univariate analysis were included in a Cox proportional hazards model with backward elimination. A *p* value < 0.05 was considered statistically significant in all analyses. Statistical analyses were conducted using SPSS version 23.0 (SPSS Inc., Chicago, IL, USA). This work has been reported in accordance with the STROCSS criteria [24].

## 3 Results

Between January 2010 and December 2020, a total of 91 liver resections were performed in 83 patients (8 of whom underwent re‐resection for liver recurrence), who constitute the study cohort. Table 1 summarizes the clinical characteristics of these 83 patients. Underlying chronic liver disease of viral etiology was documented in 12 patients (14.5%). Overall, 47 patients (56.6%) had a body mass index (BMI) ≥ 25. Of the 83 patients, 59 (71.1%) underwent major liver resection (Table 2). Twelve patients (14.5%) underwent laparoscopic liver resection. The use of the laparoscopic approach increased over time, from 8.2% (4/49) between 2010 and 2015 to 23.5% (8/34) between 2016 and 2020. This increase was statistically significant (*p* = 0.05). Fifty‐nine patients (71.1%) underwent resection for a single nodule. The distribution pattern of nodules is detailed in Table 3. Lymph node metastases were identified in 30 patients (36.1%). Sixty‐six patients (79.5%) achieved R0 liver resection.

**TABLE 1:** Characteristics of the 83 resected patients for ICC.

Variable	No. (%)/median [IQR range]
Age, years	66 [60–73]
Male sex	45 (54.2)
ASA score ≤ 2	62 (74.7)
BMI, kg/m^2^	25 [24–29]
Overweight patients (BMI 25–29.9)	34 (41.0)
Obese patients (BMI ≥ 30)	13 (15.7)
Cirrhosis	5 (6.0)
HBV	8 (9.6)
HCV	4 (4.8)
CA19‐9 (U/mL)	37.2 [10.6–142.6]
Neoadjuvant chemotherapy	10 (12.0)

Abbreviations: ASA, American Society of Anesthesiologists; BMI, body mass index.

**TABLE 2:** Operative details of the 83 resected patients for ICC.

Variable	No. (%)/median [IQR range]
Major liver resection	59 (71.1)
Laparoscopic approach	12 (14.5)
Pedicle clamping	74 (89.1)
Intraoperative blood transfusions	6 (7.2)
Postoperative complications	37 (44.6)
Severe postoperative complications	15 (18.1)
Reintervention	4 (4.8)
90‐day mortality	1 (1.2)
Hospital stay, days	9 [9–12]

**TABLE 3:** Pathologic results of the 83 resected patients for ICC.

Variable	No. (%)/median [IQR range]
Tumor size, mm	60 [60.0–87.5]
Pattern of distribution of nodules	
Type I—single	59 (71.1)
Type II—single with satellites	8 (9.6)
Type III—multifocal	16 (19.3)
AJCC 8th edition pT stage	
T1a	19 (22.9)
T1b	18 (21.7)
T2	28 (33.7)
T3	11 (13.3)
T4	7 (8.4)
AJCC 8th edition pN stage	
N0	53 (63.9)
N1	30 (36.1)
Harvested lymph nodes	5 [5–8]
Perineural invasion	14 (16.9)
Microvascular invasion	9 (10.8)
Radicality of resection	
R0	66 (79.5)
R1	17 (20.5)
Adjuvant chemotherapy	18 (22.0)

**FIGURE 1:**
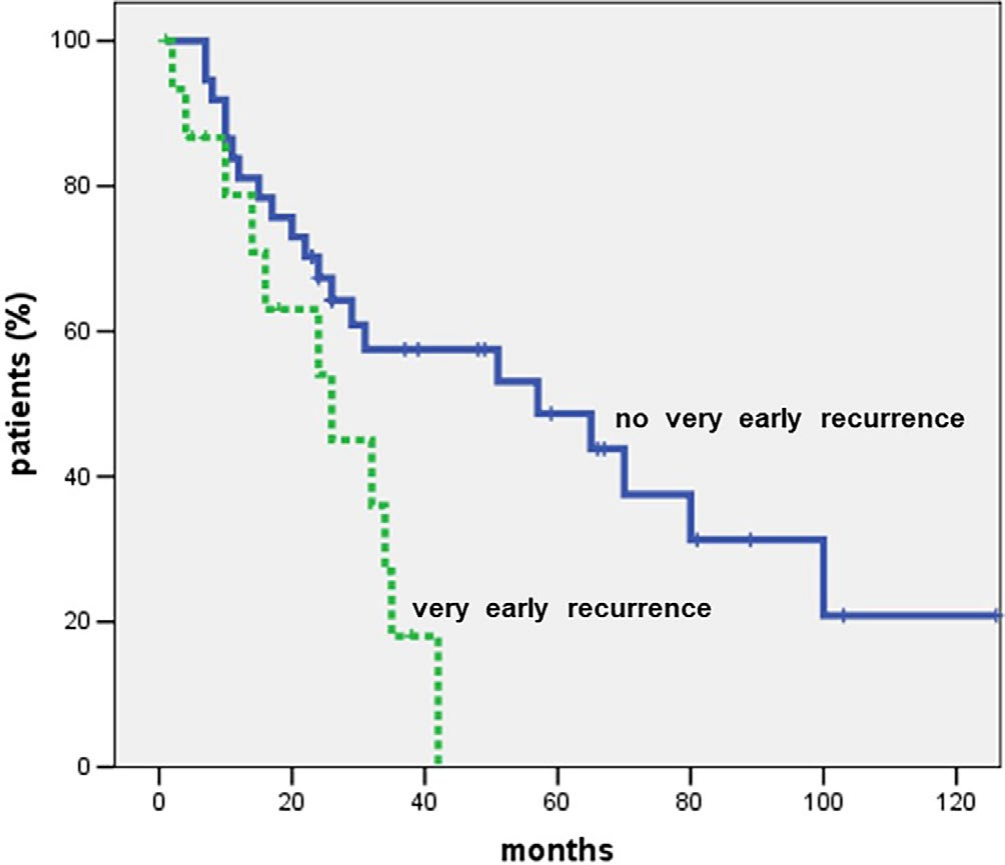
Five‐year OS according to the timing of recurrence (very early recurrence vs. no very early recurrence). Five‐year OS in patients with very early recurrence was signifi‐cantly lower than that observed in patients with no very early recurrence (0 vs. 48.7%, re‐spectively; *p* = 0.013). The median survival was 26 months in patients with very early re‐currence and 57 months in patients without very early recurrence.

After a median follow‐up of 27 months (IQR = 27–60 months), the 5‐year OS was 51.4% (median OS = 65 months). Fifty‐four patients (65.1%) experienced recurrence following liver resection. The 5‐year DFS was 33.9% (median DFS = 22 months). The pattern of recurrence is detailed in Table 4. Seventeen patients (20.5%) developed very early recurrence (within 6 months after liver resection). Among patients with recurrence within 12 months post‐resection, 69.7% (23 patients) had exclusively intrahepatic recurrence, while 30.3% (10 patients) exhibited extrahepatic recurrence with or without intrahepatic involvement. For recurrences occurring more than 12 months after surgery, 47.6% (10 patients) presented with only intrahepatic recurrence, and 52.4% (11 patients) had extrahepatic recurrence with or without intrahepatic disease. Clinical and pathological factors stratified by time of recurrence are summarized in Table 5. Timing of recurrence was significantly associated with OS; specifically, very early recurrence correlated with a markedly lower 5‐year OS compared to early or late recurrence (Figure 1). In univariate analysis, predictors of poor OS included: preoperative CA 19‐9 > 54 U/mL (*p* = 0.002); intraoperative blood transfusions (*p* = 0.030); tumor size > 5 cm (*p* = 0.008); multiple tumors (*p* = 0.003); positive lymph nodes (*p* = 0.021); R1 resection (*p* < 0.001); and occurrence of very early recurrence (*p* = 0.013). Multivariable analysis identified R1 resection (HR = 3.118; *p* = 0.003) and very early recurrence (HR = 2.891; *p* = 0.016) as independent predictors of poor OS (Table 6).

**TABLE 4:** Pattern and timing of recurrence of the 83 resected patients for ICC.

Variable	No. (%)/median [IQR range]
Pattern of recurrence	
Only intrahepatic	33 (71.1)
Only extrahepatic	7 (9.6)
Both	14 (19.3)
Timing of recurrence	
Very early (≤ 6 months)	17 (20.5)
Early (> 6 months, ≤ 12 months)	16 (19.3)
Late (> 12 months)	21 (25.3)

**TABLE 5:** Clinical and pathologic factors according to the time of recurrence.

Variable	Very early recurrence (no. 17)	Early recurrence (no. 16)	Late recurrence (no. 21)	p
Age ≥ 65 years	12 (70.6%)	5 (31.2%)	16 (76.2%)	0.611
Male sex	6 (35.3%)	13 (81.2%)	9 (42.9%)	0.768
ASA score > 2	7 (41.2%)	2 (12.5%)	3 (14.3%)	0.057
BMI ≥ 25	8 (47.1%)	12 (75.0%)	12 (57.1%)	0.241
Preop. CA 19‐9 > 54 U/mL	5 (29.4%)	7 (43.7%)	5 (23.8%)	0.364
Cirrhosis	0	1 (6.2%)	3 (14.3%)	0.096
Neoadjuvant chemotherapy	2 (11.8%)	0	2 (9.5%)	0.857
Tumor size > 5 cm	14 (82.3%)	11 (68.7%)	7 (33.4%)	0.001
Multifocal tumors	8 (47.1%)	5 (31.2%)	0	< 0.001
Intraoperative blood transfusions	2 (11.8%)	1 (6.2%)	0	0.118
Severe postoperative complications	1 (5.9%)	1 (6.2%)	4 (19.0%)	0.192
T3–T4	7 (41.2%)	1 (6.2%)	7 (33.4%)	0.762
N1	8 (47.1%)	10 (62.5%)	6 (28.6%)	0.223
Perineural invasion	3 (17.6%)	1 (6.2%)	4 (19.0%)	0.855
Microvascular invasion	3 (17.6%)	0	2 (9.5%)	0.451
R1 resection	5 (29.4%)	4 (25.0%)	3 (14.3%)	0.266
Adjuvant chemotherapy	3 (17.6%)	3 (18.7%)	5 (23.8%)	0.794

**TABLE 6:** Univariate and multivariable analysis of OS in 83 resected patients for ICC.

Variable	Univariable analysis		*p*	Multivariable analysis		*p*
	No. (%)	5‐year OS		HR	95% CI	
Age (years)			0.713			
< 65	36 (43.4)	42.7				
≥ 65	47 (56.6)	57.5				
Sex			0.988			
Male	45 (54.2)	55.3				
Female	38 (45.8)	46.2				
ASA score			0.963			
≤ 2	62 (74.7)	52.2				
> 2	21 (25.3)	49.1				
BMI (kg/m^2^)			0.867			
< 25	36 (43.4)	51.7				
Overweight/obese patients (≥ 25)	47 (56.6)	49.8				
Viral hepatitis			0.552			
Yes	12 (14.5)	62.5				
No	71 (85.5)	49.9				
Preop. CA 19‐9 > 54 U/mL						
Yes	22 (26.5)	22.1	0.002			
No	61 (73.5)	64.8				
Major liver resection			0.066			
Yes	59 (71.1)	45.7				
No	24 (28.9)	64.5				
Laparoscopic approach			0.605			
Yes	12 (14.5)	64.6				
No	71 (85.5)	50.0				
Intraoperative blood transfusions			0.030			
Yes	6 (7.2)	0				
No	77 (92.8)	53.8				
Severe postoperative complications			0.573			
Yes	15 (18.1)	67.7				
No	68 (81.9)	37.7				
Tumor size			0.008			
≤ 5 cm	39 (47.0)	72.8				
> 5 cm	44 (53.0)	29.0				
No. of tumors			0.003			
Single	59 (71.1)	61.2				
Single with satellites	8 (9.6)	28.6				
Multifocal	16 (19.3)	26.9				
T stage			0.119			
T1–T2	65 (78.3)	54.3				
T3–T4	18 (21.7)	32.8				
*N* stage			0.021			
N0	53 (63.9)	55.6				
N1	30 (36.1)	41.1				
Perineural invasion			0.267			
Yes	14 (16.9)	46.2				
No	69 (83.1)	53.0				
Microvascular invasion			0.343			
Yes	9 (10.8)	33.3				
No	74 (89.2)	53.3				
Radicality of resection			< 0.001	3.118	1.467–6.627	0.003
R0	66 (79.5)	62.2				
R1	17 (20.5)	9.6				
Timing of recurrence						
Very early vs. no very early			0.013	2.891	1.214–6.884	0.016
Very early	17 (31.5)	0				
No very early	37 (68.5)	48.7				
Very early vs. early vs. late			< 0.001			
Very early	17 (31.5)	0				
Early	16 (29.6)	15.6				
Late	21 (38.9)	70.9				
Adjuvant chemotherapy			0.762			
Yes	18 (21.7)	63.2				
No	65 (78.3)	54.7				

In univariate regression analysis, a CA 19‐9 level > 54 U/mL was significantly associated with an increased likelihood of recurrence (OR 4.817; 95% CI 1.203–19.285) (Table 7). In multivariable regression analysis, tumor size > 5 cm was independently associated with a higher risk of very early recurrence (OR 5.721; 95% CI 1.236–26.486), while a BMI ≥ 25 was associated with a lower risk of very early recurrence (OR 0.223; 95% CI 0.055–0.901) (Table 8).

**TABLE 7:** Univariate analysis of preoperative, intraoperative and postoperative factors associated with recurrence after liver resection.

Variable	Univariable analysis *p*	OR (95% CI)
Age ≥ 65 years	0.157	
Male sex	0.529	
ASA score > 2	0.467	
BMI ≥ 25	0.445	
Preoperative CA 19‐9 > 54 U/mL	0.026	4.817 (1.203–19.285)
Cirrhosis	0.522	
Neoadjuvant chemotherapy	0.067	
Tumor size > 5 cm	0.118	
Multifocal tumors	0.177	
T3‐T4	0.093	
N1	0.087	
Perineural invasion	0.409	
Microvascular invasion	0.457	
R1 resection	0.700	
Adjuvant chemotherapy	0.676	

**TABLE 8:** Multivariable analysis of preoperative, intraoperative and postoperative factors associated with very early recurrence.

Variable	Univariable analysis *p*	Multivariable analysis OR (95% CI)	*p*
Age ≥ 65 years	0.336		
Male sex	0.104		
ASA score > 2	0.029		
BMI ≥ 25	0.037	0.223 (0.055–0.901)	0.035
Preoperative CA 19‐9 > 54 U/mL	0.764		
Cirrhosis	0.999		
Neoadjuvant chemotherapy	0.418		
Tumor size > 5 cm	0.026	5.721 (1.236–26.486)	0.026
Multifocal tumors	0.029		
T3–T4	0.158		
N1	0.793		
Perineural invasion	0.692		
Microvascular invasion	0.171		
R1 resection	0.392		
Adjuvant chemotherapy	0.712		

According to the preoperative very early recurrence score described by Tsilimigras et al. [14], 77 patients (92.8%) were classified as low risk, 5 patients (6.0%) as intermediate risk, and 1 patient (1.2%) as high risk. Five‐year OS differed significantly among the three groups: 51.0% in the low‐risk group, 8.0% in the intermediate‐risk group, and 0% in the high‐risk group (*p* = 0.047). The rate of very early recurrence was 21.0% (16/77) in low‐risk patients, 20.0% (1/5) in intermediate‐risk patients, and 0% in high‐risk patients. Five‐year recurrence‐free survival (RFS) rates, however, were not significantly different across the groups: 34.7% (low risk), 40.0% (intermediate risk), and 0% (high risk) (*p* = 0.788). The preoperative scoring system stratified patients into three groups based on scores of 0–3, 4–5, and 6–9. The corresponding 5‐year RFS rates were 40.6%, 24.7%, and 20.0%, respectively, with no statistically significant difference (*p* = 0.124). Using the postoperative very early recurrence score, 61 patients (73.5%) were classified as low risk, 14 (16.9%) as intermediate risk, and 8 (9.6%) as high risk. Five‐year OS significantly differed among these groups: 40.0% for low risk, 34.1% for intermediate risk, and 0% for high risk (*p* = 0.006). The rates of very early recurrence were 18.0% (11/61) in the low‐risk group, 28.6% (4/14) in the intermediate‐risk group, and 25.0% (2/8) in the high‐risk group. Five‐year RFS rates showed a trend toward difference but did not reach statistical significance: 39.5% (low risk), 17.5% (intermediate risk), and 0% (high risk) (*p* = 0.062). The postoperative scoring system also categorized patients into three groups based on scores of 0–4, 5–6, and 7–10. The corresponding 5‐year RFS rates were 39.5%, 17.5%, and 0%, respectively, without reaching statistical significance (*p* = 0.062).

## 4 Discussion

This study confirmed that curative liver resection for ICC can be achieved in approximately 80% of selected patients (rate of R0 resections: 79.5%). The perioperative outcomes were acceptable, with a 90‐day mortality rate of 1.2% and a severe complication rate of 18.1%. The majority of patients (71.1%) underwent major hepatectomies, reflecting the typically advanced stage of ICC at presentation. The median tumor size was 6 cm, and the cohort showed a high prevalence of overweight and obesity (41.0% and 15.7%, respectively), consistent with recognized ICC risk factors [25]. Long‐term outcomes were comparable to existing literature, with a 5‐year overall survival (OS) rate of 51.4% [25]. Importantly, R1 resection and very early recurrence (within 6 months) were identified as independent predictors of poor OS (HR = 3.118, *p* = 0.003; HR = 2.891, *p* = 0.016). Recurrence occurred in 65.1% of patients, and 20.5% experienced very early recurrence, which was associated with a 0% 5‐year OS. Predictive factors for recurrence included preoperative CA 19‐9 > 54 U/mL and predictive factors for very early recurrence included tumor size > 5 cm. The prognostic scoring system by Tsilimigras et al. [14] was validated, but its practical utility was limited due to the retrospective selection bias and the predominance of resected patients who belong to the low‐risk group (92.8%).

The findings emphasize the critical prognostic role of resection margin status [26, 27] and recurrence timing [28]. Achieving R0 resection remains essential for improving long‐term survival, and careful patient selection is key. However, the high rate of very early recurrence (21.0%) even among patients classified preoperatively as low risk suggests that conventional clinicopathological criteria are insufficient to guide surgical decisions.

Given the limitations of clinical predictors, the study supports the integration of radiomics features and molecular data to enhance prognostication. Preoperative CT‐based radiomics models and molecular subtyping have demonstrated promise in identifying patients at high risk for recurrence [29, 30, 31]. These tools could help refine treatment algorithms, identify candidates for neoadjuvant therapy, or consider alternative strategies in borderline cases. Moreover, metrics like sarcopenia may further aid in developing tailored approaches that move beyond one‐size‐fits‐all surgery [32].

A key strength of this study lies in its comprehensive analysis of recurrence dynamics and prognostic factors within a relatively large and well‐characterized cohort. However, its retrospective design introduces potential selection bias, especially regarding the preoperative risk stratification. The limited proportion of patients classified as intermediate‐ or high risk preoperatively may have reduced the generalizability of the Tsilimigras score in real‐world settings. Future prospective studies should incorporate radiomics, molecular, and functional parameters (e.g., sarcopenia, performance status) to develop more robust predictive models. Research should also explore the potential benefit of neoadjuvant therapies in high‐risk subsets to mitigate very early recurrence.

## 5 Conclusions

In summary, approximately one in five patients undergoing curative‐intent liver resection for ICC experiences very early recurrence, which is strongly associated with dismal long‐term outcomes. Conventional clinical parameters alone are inadequate for guiding treatment decisions in this population. Incorporating radiomics, molecular, and functional markers holds the potential to improve risk stratification, personalize care, and ultimately enhance survival in resectable ICC.

## Author Contributions

**Francesco Ardito:** conceptualization, methodology, project administration, supervision, writing – review and editing. **Francesco Razionale:** data curation, formal analysis, investigation, writing – original draft. **Andrea Campisi:** data curation, formal analysis, investigation, writing – original draft. **Çınar Turgay:** data curation, formal analysis, investigation, writing – original draft. **Alessandro Coppola:** methodology, validation, writing – original draft, writing – review and editing. **Simone Vani:** data curation, formal analysis, resources, software. **Maria Vellone:** conceptualization, supervision, validation, writing – original draft, writing – review and editing. **Felice Giuliante:** conceptualization, supervision, validation, writing – review and editing.

## Conflicts of Interest

The authors declare no conflicts of interest.
